# Quantum dot-integrated GaN light-emitting diodes with resolution beyond the retinal limit

**DOI:** 10.1038/s41467-022-29538-4

**Published:** 2022-04-06

**Authors:** Junho Bae, Yuseop Shin, Hyungyu Yoo, Yongsu Choi, Jinho Lim, Dasom Jeon, Ilsoo Kim, Myungsoo Han, Seunghyun Lee

**Affiliations:** 1 Department of Electronic Engineering, Kyung Hee University, Yongin, 17104 Republic of Korea; 2 Department of Electronics and Information Convergence Engineering, Kyung Hee University, Yongin, 17104 Republic of Korea; 3 LG Display Research and Development Center, Seoul, 07796 Republic of Korea

**Keywords:** Electrical and electronic engineering, Inorganic LEDs

## Abstract

Near-eye display technology is a rapidly growing field owing to the recent emergence of augmented and mixed reality. Ultrafast response time, high resolution, high luminance, and a dynamic range for outdoor use are all important for non-pixelated, pupil-forming optics. The current mainstream technologies using liquid crystals and organic materials cannot satisfy all these conditions. Thus, finely patterned light-emissive solid-state devices with integrated circuits are often proposed to meet these requirements. In this study, we integrated several advanced technologies to design a prototype microscale light-emitting diode (LED) arrays using quantum dot (QD)-based color conversion. Wafer-scale epilayer transfer and the bond-before-pattern technique were used to directly integrate 5-µm-scale GaN LED arrays on a foreign silicon substrate. Notably, the lithography-level alignment with the bottom wafer opens up the possibility for ultrafast operation with circuit integration. Spectrally pure color conversion and solvent-free QD patterning were also achieved using an elastomeric topographical mask. Self-assembled monolayers were applied to selectively alter the surface wettability for a completely dry process. The final emissive-type LED array integrating QD, GaN, and silicon technology resulted in a 1270 PPI resolution that is far beyond the retinal limit.

## Abstract (web summary)

Augmented reality technologies typically rely on near-eye displays, which requires displays with very high resolutions. Here, Bae et al demonstrate a quantum-dot integrated GaN light emitting diode arrays with a pixels per inch resolution of 1270, well beyond the retinal limit.

## Introduction

Displays beyond eye-limiting resolution have attracted substantial interest as a key enabler for augmented/virtual reality (AR/VR) and ultrahigh-resolution (e.g., 8 K and 16 K) screens. A near-eye device utilized in a head-mounted display (HMD) is located in close proximity to the pupil, which inevitably requires a significant leap in pixel resolution and response speed^1–16^. Next-generation displays for industrial, medical, and outdoor uses also require sub-10 μm pixels with short response times and high optical contrast for flicker-free imaging and minimal screen-door effects.

Liquid crystal displays (LCD) that dominate the current market are a transmissive type of display with inevitably lower energy efficiencies and optical contrast compared with self-emissive technology owing to their inherent limitations^3,11,17,18^. The pixel density of the LCDs is also limited by their low-resolution production methods. Organic LEDs (OLEDs), an emissive technology, are superior in terms of efficiency and dynamic range compared with LCDs^18^. For OLEDs, the intrinsic susceptibility of organic polymers for solvent dissolution was a major challenge in reaching such a high-pixel density because direct lithography required solvents. Hence most commercial OLEDs relied on shadow masking technologies such as the fine metal mask method in the past. However, recent development in OLED patterning technologies has led to the development of displays with exceedingly high resolution. Joo et al., for example, utilized nanopatterned metasurface mirrors based on nanoimprinting to achieve several 10 s of nm-sized pixels and over 10,000 PPI^19^. Haas et al. also achieved full-color OLEDs with a pixel size of 10 µm^20^. Regarding the response speed, it is worthwhile to note that the perceived speed of a display is a combinational effect of many factors (more discussion in the Supplementary Information discussion 1). Nonetheless, the modest latency associated with the limited response time of liquid crystals, organic polymers, and thin-film transistors is still a challenge^5,7,10,11^. Moreover, organic materials still suffer from electroluminescence degradation and lifetime issues in high-luminescence outdoor conditions^8,18,21,22^.

One solution to this dilemma is the use of advanced microfabrication techniques with inorganic solid-state devices and integrated complementary metal-oxide-semiconductor (CMOS) circuits. With the constant demand for high energy efficiency, long lifetime, and high luminosity, micro-LED displays based on inorganic semiconductors, such as GaN, are promising candidates for ultrahigh-resolution displays^5,6,8,12,18,23^. Recent advances in flip-chip bonding of the GaN epitaxial layer with silicon wafers have also opened up doors for CMOS integration^2,24^. However, miniaturization of individual pixels to a few μm in terms of feature size comes at a significant cost and results in several drawbacks.

**Fig. 1:**
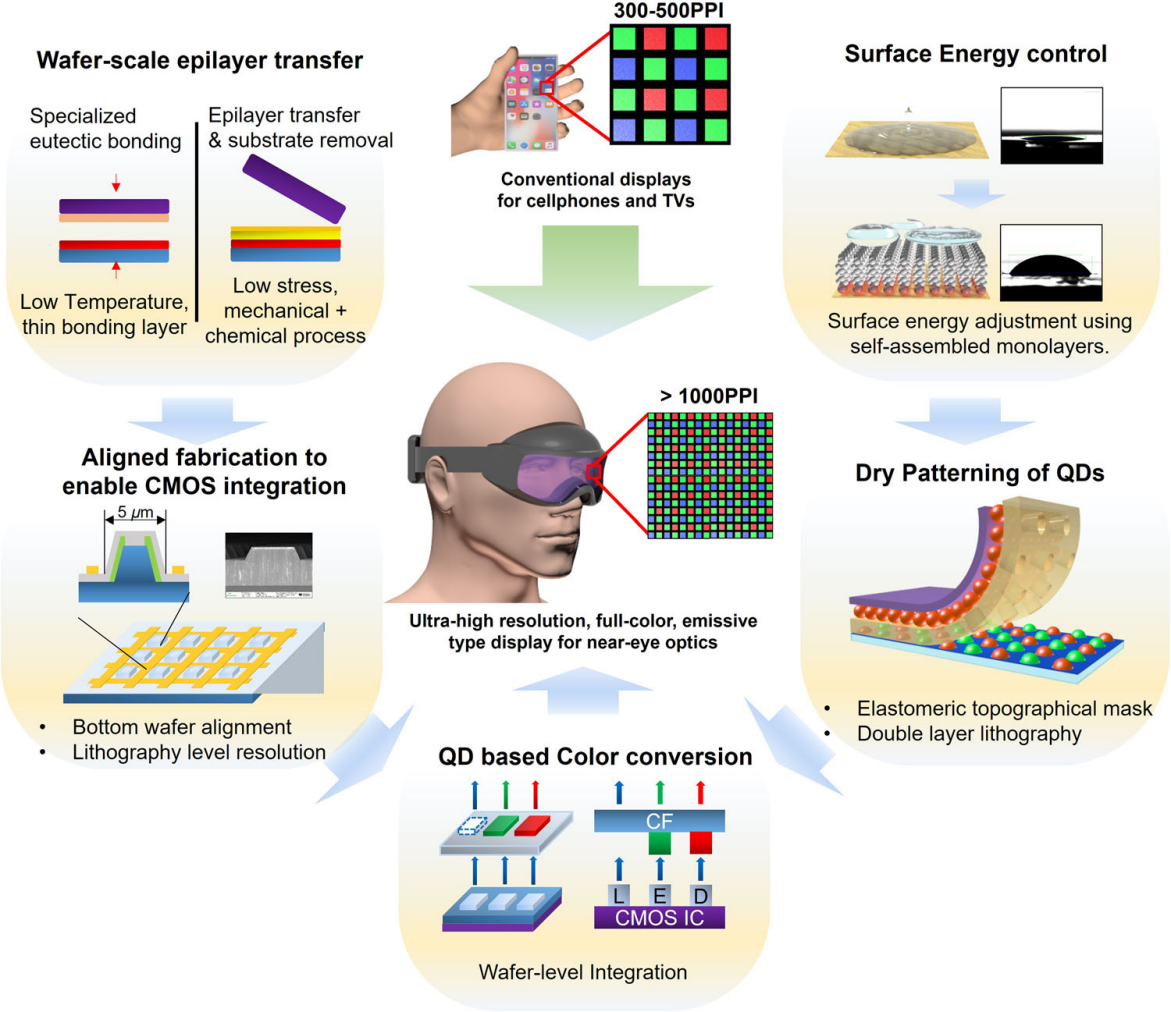
An overview of the key technologies used in this study to achieve an emissive-type RGB micro-LED array with over 1000-PPI resolution. **a** Wafer-scale GaN epilayer was transferred using a low temperature bonding process. **b** The epilayer was bonded with lithographic precision in alignment for future CMOS integration. **c** Surface energy was modified with self-assembled monolayers for dry patterning of QDs. **d** A double layer elastomeric mask was used to dry pattern the QDs without using any solvent. **e** QDs were used for spectrally pure color conversion.

An important goal of this study was to address the two most difficult challenges and, in doing so, provide an innovative solution to building full-color, emissive-type displays with beyond eye-limiting resolution (Fig. 1). First, technical challenges associated with surface warping, pixel resolution, and large-scale array formation must be overcome. Efficient direct bandgap materials for light emission, such as GaN, are typically grown on either a sapphire or a silicon wafer. Unfortunately, severe warping of the wafer surface is common owing to the residual thermal stress and lattice mismatch between materials^25–29^. Even if successful lithography is conducted and small pixels (<10 μm) are patterned, aligning such miniature features to another silicon wafer during the bonding process is quite challenging^30^. Usually, a wafer-bonding technique involves mechanical alignment between two wafers and applying pressure in a heated environment. There are many structural, mechanical difficulties involved in this process (stress, warped surfaces, thermal effects, and step height difference) that can cause the wafers to misalign. The degree of misalignment depends on different equipment and techniques. However, aligning a sub-10 μm LED is consensually thought to be challenging because the misalignment should be limited to 100s of nm or lower.

Hence, the conventional method of forming μm-scale GaN LEDs on the original substrate and transferring the epitaxial LED layer to another silicon substrate (possibly with a CMOS circuit) is impractical. Individual pixel transfer methods, flip-chip bonding, or roll-to-roll methods are also not compatible with this feature size. Here we overcame the alignment problem by developing an extremely thin bonding alloy and using a bond-before-pattern technique. When the wafer is bonded first before patterning the LEDs, many of the problems are mitigated because the consecutive lithography on the top epilayer is conducted with the alignment mark from the bottom wafer. Hence, there will be a lithography-level alignment between the top and the bottom substrates. A strong and thin wafer-bonding alloy layer that allows etching using a plasma etching method is crucial in this approach to form an electrical connection between the top and bottom layers. A stable GaN substrate removal/etching technique allows the relaxation of residual stress in the GaN epitaxial layer and subsequent formation of GaN LEDs on the new substrate without the alignment issue.

In the past, there have been efforts to transfer the epitaxial III–V layer to another silicon substrate similar to this work’s approach. JBD company, for example, transferred GaN epilayer from the sapphire substrate to another Si IC wafer and successfully fabricated the GaN LED arrays on top^31^. Notably, while they used GaN-on-sapphire wafers, our work is focused on transferring epilayer from the GaN-on-Si wafers. Both approaches have their pros and cons. GaN-on-sapphire is a more mature technology and readily available. However, a sapphire substrate is quite expensive and the thermal expansion coefficient of sapphire (5.8–7.7 × 10^−6^ K^−1^) is significantly larger than that of GaN (3.17 × 10^−6^ K^−1^). GaN-on-Si, on the other hand, utilizes a more cost-effective Si substrate (2.6–3.2 × 10^−6^ K^−1^) with less difference in thermal expansion coefficient compared to that of GaN. Consequently, less wafer bowing was observed on GaN-on-Si compared to GaN-on-sapphire (Supplementary Fig. S1). Experimentally, thermal residual stress on the GaN epilayer had a significant effect on the bowing of the whole wafer and the yield was superior with GaN-on-Si wafers. Quesnel et al., for example, also fabricated LED microdisplays based on GaN-on-Si technology mentioning the manufacturability of such an approach^32^.

Second, successful implementation of a high-quality, full-color display requires red, green, and blue (RGB) color conversion with high spectral purity and a wide color gamut. Numerous studies have been conducted using new materials as color conversion layers. Among the various candidates, quantum dots (QD) are known to have a strong potential owing to their narrow emission spectra, extensive color tenability, and high quantum yield^33–43^. For conventional QD patterns, various transfer printing methods with fine metal masks^44–47^ and inkjet printing^48–50^ can be employed. However, these techniques have limitations in achieving a uniform array with ultrahigh-resolution and high fidelity. Lithography is an obvious solution to the resolution issue. Nevertheless, the majority of color conversion materials, including QDs, are sensitive to organic solvents^51–54^, and direct lithography patterning of photoresists on top of a QD layer is a challenge. More importantly, the luminous properties of QDs often degrade to an insignificant degree after patterning with lithography, which is a decisive disadvantage. There are alternative methods similar to lithography that can result in high resolution. Yang et al. achieved a QD minimum feature size of 3 μm by applying QD blended materials using a UV light-driven ligand crosslinker (LiXer)^55^. The advantage of this method is that the robust bonding between QD particles and ligands can withstand consecutive processes to form RGB QDs. One drawback is that each patterned QD layer had a thickness between 15 and 30 nm. Hence, the author wrote that repeated cycles of QD film deposition, photo-patterning is necessary to reach at least a μm-thick layer suitable for color conversion. Li et al. achieved a pixel size of 6 um by using a QDs/thiol-ene photo-polymer composite with a UV digital light processing (DLP) printer projection lithography^56^. The advantage is that QDs with amine surface groups are uniformly dispersed, which reduces aggregation and improves conversion efficiency compared to drop-cast QDs. However, patterning was achieved by rinsing the uncured composite with IPA, which means that the QDs were still exposed to solvents. Here, we present a unique hybrid technology of transfer printing µm-scale QDs by exploiting the surface energy differences of various interfaces that were treated with self-assembled monolayers. Elastomeric topological masks and double-layer photolithography were fully utilized to pattern the QDs with no exposure to any liquid. Importantly, this method enables direct patterning of QDs on a wafer-scale substrate; the QD layer is sufficiently thick for highly efficient color conversion.

## Results

### Wafer-scale epilayer transfer and μ-LED formation

**Fig. 2:**
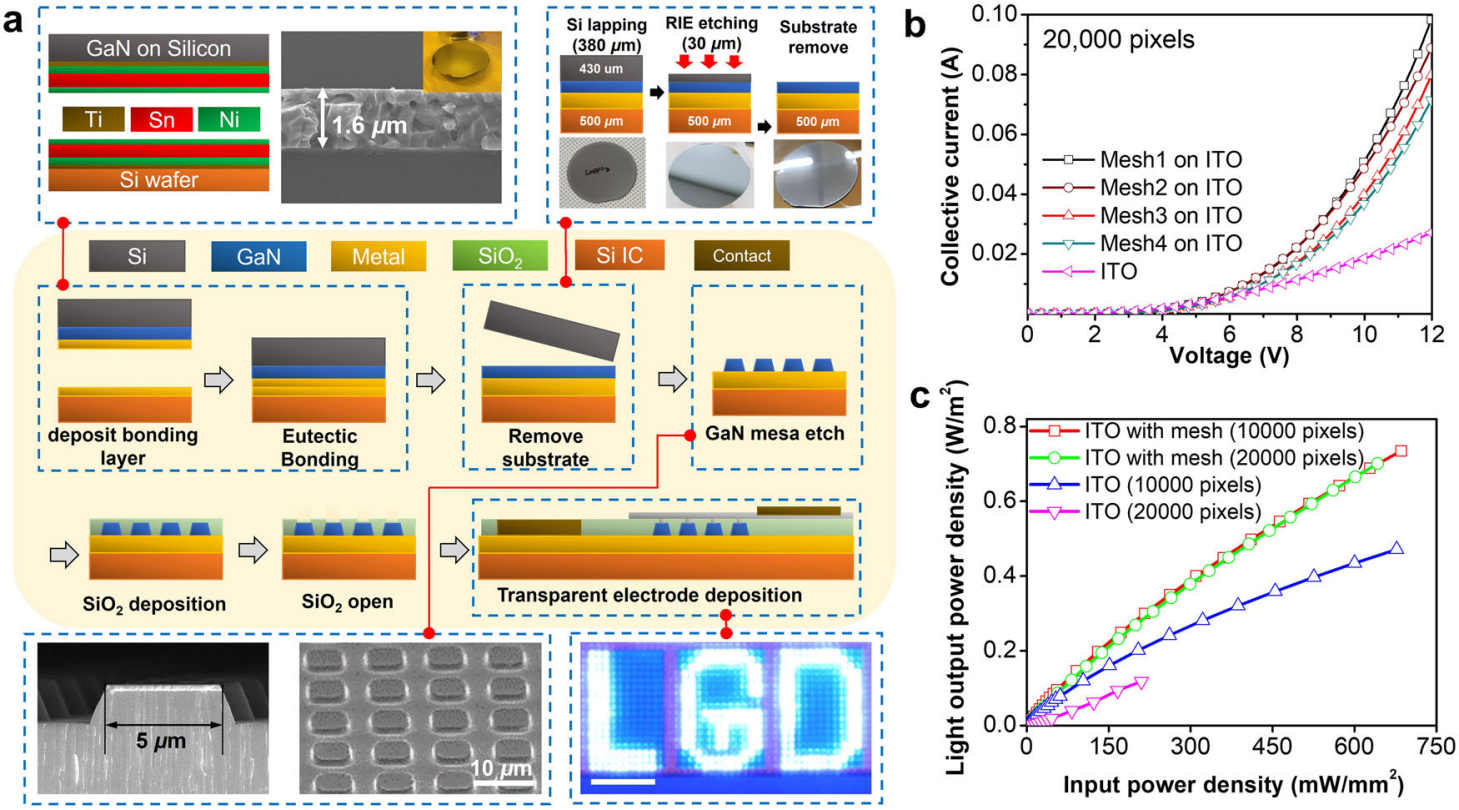
Fabrication of an epitaxial layer-transferred μ-LED and measurement results. **a** Schematic of wafer-scale epitaxial layer transfer and the μ-LED fabrication process. A cross-sectional SEM image of bonded eutectic metal alloys (without any voids) is shown on the top left. Schematic images and optical images of the substrate removal process is shown in top right. FE-SEM images of 5 μm-pixel μ-LED mesa structures and an optical image of LGD letter patterns with individual pixels shown are on the lower panel. **b** Collective current–voltage characteristics of 20,000-pixel μ-LEDs with varying metal mesh densities. **c** Light output power densities of μ-LED structures as a function of input power.

The process of delaminating the GaN epitaxial layer from the original substrate and bonding it to a different silicon substrate is presented in Fig. 2. One important goal of this process is to demonstrate that the GaN epitaxial layer can be transferred to an arbitrary substrate and the LEDs formed with high alignment accuracy with the new substrate. Our successful demonstration will confirm that LEDs can be formed on top of a silicon wafer with CMOS circuitry and that individual pixels can be directly controlled by the underlying transistors.

One important issue at this stage is the unpredictable warping of the GaN substrate caused by the lattice mismatch and thermal expansion difference between materials. While photolithography is a highly effective method for patterning on flat surfaces, substrates that are topologically irregular create various problems in terms of alignment, reproducibility, and yield.

The warpage level of the GaN substrates from general industries is on the order of a few 10 s of μm to 200 μm, depending on the substrate size and material (Supplementary Fig. S1a). A 4 inch GaN on silicon was used in this study as the silicon substrate had less warpage (~20 μm from the center to the edge, Supplementary Fig. S1b), and the micromachining was less complicated. However, even such a low warpage can be critical for sub-5 μm patterns, and relaxation of the residual stress on the epitaxial layer is crucial for the device yield. Instead, of using a conventional method of transferring prefabricated devices, fabricating the devices after wafer-bonding and epilayer transfer can dramatically improve the alignment and patterning issues (Supplementary Fig. S1c). A low-temperature, low-stress bonding technique with a thin (<5 μm) bonding layer and the development of a reliable substrate removal process are extremely important to achieve an accurate alignment with the bottom silicon wafer. A thin bonding layer that can be readily etched is important to form an individual through-bias between the top LED pixels and the new substrate. The bonding material has to be sufficiently thick for high mechanical strength but thinner than the diode width to prevent device skew. To meet these requirements, a bonding alloy composed of Ti, Sn, and Ni was deposited via e-beam evaporation (Fig. 2a). The use of In and Au as a bonding material was avoided to prevent thermal diffusion, void formation, and high manufacturing costs^57^. The details of the Ti, Sn, and Ni eutectic bonding process are provided in the supplementary information (Supplementary Fig. S2). Once the two wafers were attached, a wet etching process using a hydrofluoric, nitric, and acetic (HNA) solution was employed to remove the bulk of the silicon substrate from the GaN on the Si wafer^58,59^. The other substrate was passivated with a SiN coating so that it was protected from the corrosive wet etchant. The subsequent lapping and reactive ion etching (RIE) process formed a smooth and flat GaN epilayer (Fig. 2a). Subsequently, 5 μm-wide mesa structures were then etched with an RIE process using Cl and BCl_3_ to pattern the LEDs (Fig. 2a). Detailed process techniques are provided in the supplementary information (Supplementary Fig. S3).

Upon LED formation, the resistance of the transparent electrode was found to be critical to the formation of a large, continuous LED array. The voltage drops across the transparent electrode are parasitic issues that reduce the overall efficiency. This effect is more pronounced with GaN LEDs as they require a relatively low voltage drop across the diode compared with liquid crystals and organic LEDs. The numerous LEDs in the array also decrease the voltage drop across the diodes as it forms a massively parallel resistance array. Hence, exploiting a hybrid transparent conductor with exceptionally low sheet resistance by using indium tin oxide (ITO) and a metallic mesh structure was found to be important (Fig. 2b, c)^60^. Meshes 1, 2, 3, and 4 correspond to mesh densities of 1, 4, 9, and 16 pixels in a grid, respectively, (Supplementary Fig. S3). We confirmed that the electrical properties were improved with the application of a hybrid-mesh conductor compared with a stand-alone ITO (Fig. 2a, b, and Supplementary Fig. S3g). Such an effect was more pronounced with a 20,000-pixel panel compared to that of a 10,000-pixel panel because the former has a larger array area and LEDs are farther away from the contact. Hence light extraction efficiency was more susceptible to the higher sheet resistance of the ITO when the array was larger (Fig. 2b, c). Interestingly, mesh design 2 (four pixels per grid) resulted in the highest efficiency (light output power density per driving current) in terms of light output compared with other designs (Supplementary Fig. S3g) even though mesh design 1 exhibited the highest current per input voltage due to its low overall sheet resistance. This outcome was attributed to the trade-off between the light blockage and the decrease in sheet resistance due to the metal meshes, which was previously reported^60,61^. This may be counterintuitive because the mesh is not directly above the LED and it does not seem to block the light. However, light extraction is not always vertical to the surface because the GaN LEDs are typically tapered due to the etch profile of the GaN layer. Moreover, GaN light sources are Lambertian type and some parts of the light are either absorbed or reflected in different directions due to the interaction between various surfaces and interfaces^62^.

### Surface energy control and elastomer mask-assisted quantum-dot patterning

**Fig. 3:**
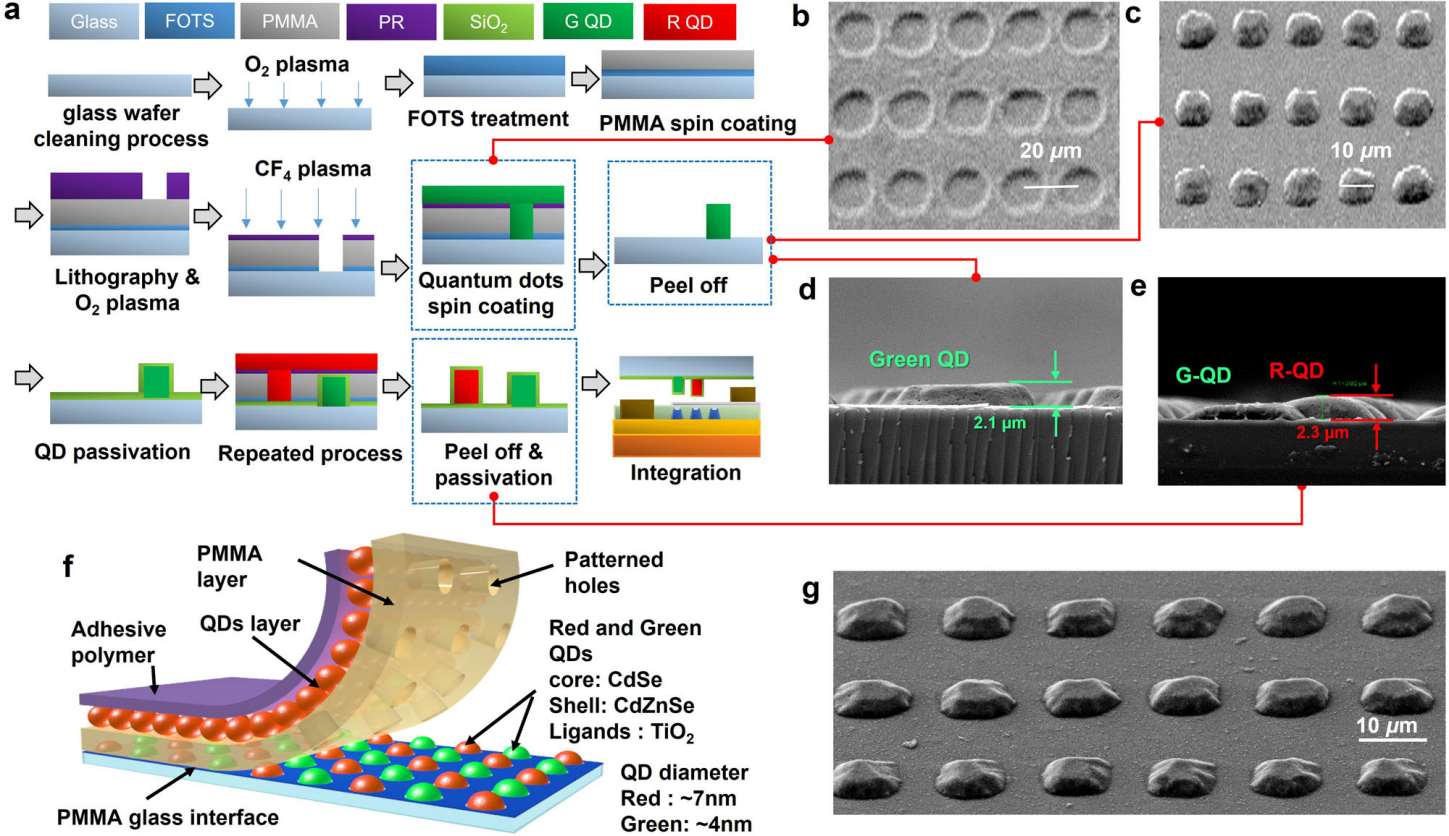
Patterning of QD films using the elastomer-assisted dry-patterning process. **a** A schematic of the overall process of dry patterning of microscale QD pixels. **b** QD layer before the peel-off process. **c** Patterned individual QD pixels. **d** SEM image of a QD array with a single**-**color QD pattern. **e** SEM image of a QD array with both QDs sequentially patterned. **f** Illustration of the elastomeric mask used for QD dry patterning. **g** Isotropic SEM image of the patterned QD.

Patterning QDs into sub-10 µm pixels is an important but exceedingly difficult task. Similar to organic materials, the quality of QDs degrades significantly upon contact with most chemicals, including solvents and water. Thus, general photolithography, which is one of the few ways to pattern sub-10 µm features with precise alignment, is not compatible with QDs. The fine metal mask (FMM) method and inkjet printing are the most common methods for quantum-dot patterning. However, the FMM method requires periodic cleaning and has a micro-forming problem due to limitations in metal mask thickness^44–47^. Although there has been a significant development in inkjet printing technology, nozzle clogging is a common issue^48–50^. More importantly, both of these techniques cannot reliably pattern sub-10 µm features. To solve this, a hybrid approach that incorporates the advantages of both lithography and the shadow mask method was developed (Fig. 3). To produce large-scale, high-resolution QD patterns, we used a photo-patternable elastomer-based mask inspired by dry lift-off technology, a technique for forming ultrafine metal patterns using surface energy adjustment^63–72^. The application of this technology requires an understanding of the characteristic surface energy, solvent polarity, and fluoridation of polymers.

The control of the ultimate QD pattern size and thickness begins with careful adjustment of the surface energy using self-assembled monolayers (SAM). One effective and simple method to selectively control the surface energy of a glass substrate is to use a SAM called perfluorooctyl trichlorosilane (FOTS, Sigma–Aldrich Co.), as presented in Fig. 3a. Interestingly, the conventional FOTS coating method for using vapor-phase treatment under vacuum^71,73,74^ was not applicable as the resulting surface energy was too low for spin-coating the polymethyl methacrylate (PMMA)-supporting structure (Supplementary Fig. S4). More importantly, the commonly used PMMA with anisole as the solvent was not suitable as the polarity of anisole was too high to be coated on the FOTS-treated surface. After multiple experiments, drop-casting FOTS for 2 min and spin-coating PMMA dissolved in a non-polar solvent, such as toluene^75^, at a 3-wt./vol% concentration was found to be the most effective way to form the PMMA layer. Thus, maintaining an appropriate level of surface energy that is appropriate for both spin-coating and peeling off of the PMMA layer was crucial.

As presented in Fig. 3a, a photoresist layer was coated on top of the cured PMMA to conduct conventional photolithography. Subsequent O_2_ plasma was utilized to etch the bottom PMMA layer. Then, the QD layer was deposited before the final peel-off process. Normally, one would expect a direct deposition of QDs, and peeling of the bottom structural layer would be a straightforward process to form reliable QD patterns. However, the overall process required much more attention to the material interfaces. As expected, a trade-off between the QD layer thickness and QD pattern size was required. A thicker QD is generally required for an efficient color conversion. However, smaller patterns result in less-adhesive force between the QD and the bottom substrate. The adhesive force of the PMMA sidewall was also quite high for the QDs to leave a dependable layer attached to the bottom substrate after the peel-off process. As fluoropolymers have weaker adhesive properties, CF_4_ treatment was found to be highly effective in increasing the yield and thickness of the QD patterns.

For green or red color conversion, QDs with a CdSe core and CdZnSe shell structure with TiO_2_ ligands were utilized. The QD diameters for green and red light conversion were 4 and 7 nm, respectively (Fig. 3f). To form a double pattern with both green and red QDs, first, green QDs were formed and then cured at both 90 °C and 180 °C for 2 min, respectively, (Supplementary Fig. S7). SiO_2_ was then deposited as a passivation layer before the formation of red QD. SiO_2_ was deposited using a gentle room temperature physical vapor deposition method and had little effect on the underlying green QDs, which were already cured at 180 °C. SiO_2_ was used as its surface energy was found to be similar to that of a glass substrate, and FOTS was quite effective. A double-patterned QD array is presented in Fig. 3e. The shape and the corresponding height profile were also confirmed via an isotropic SEM image, as presented in Fig. 3g.

### Analysis of the QD-integrated micro-LED display

**Fig. 4:**
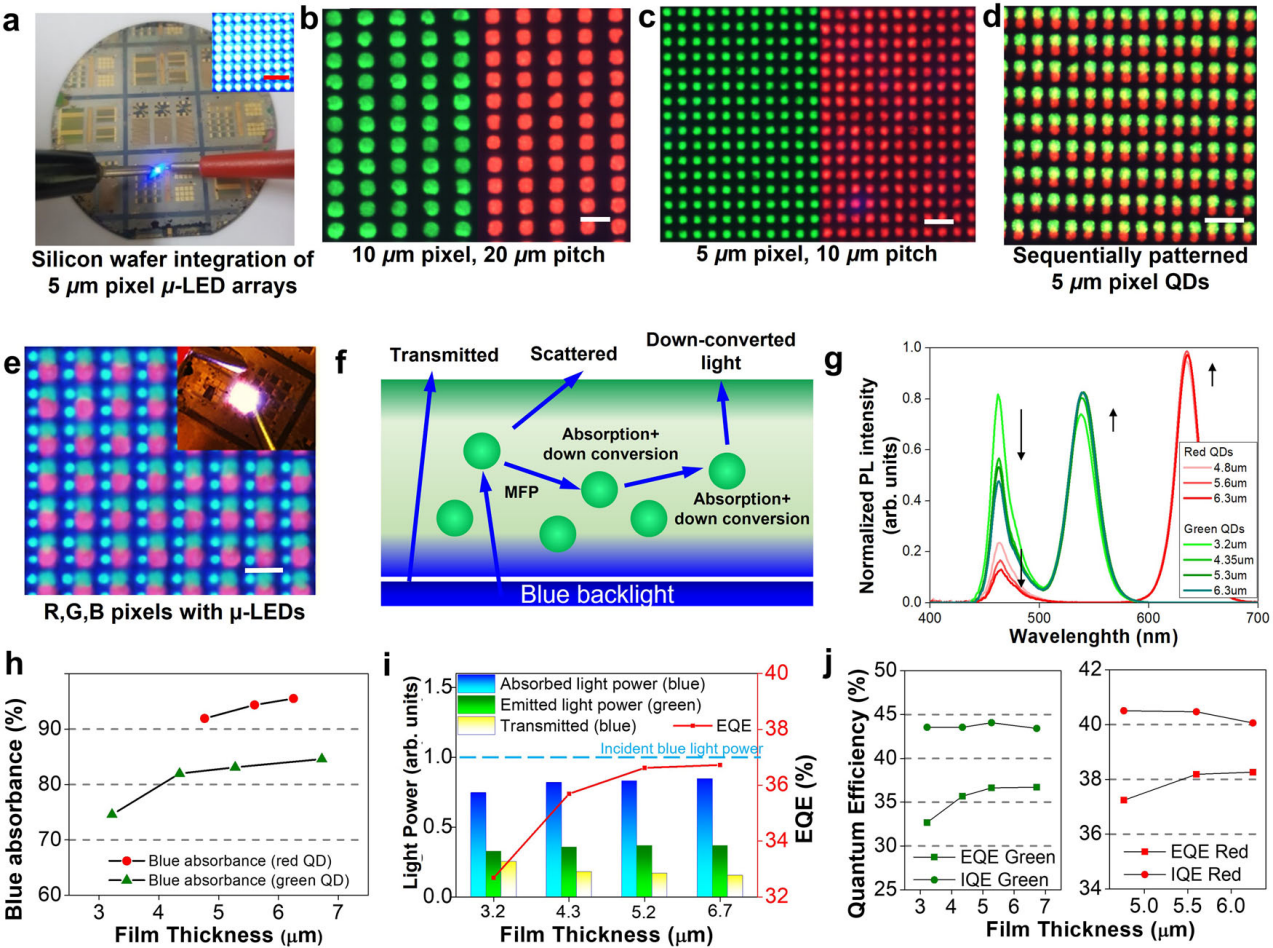
Characterization of the QD-integrated full-color μ-LED display. **a** Photographic image of the wafer with an ultrafine pixel micro-LED array. **b**, **c** Microscopic images of the R/G single QD patterns under UV light with 10 and 5 μm pixels, respectively. All scale bars denote 20 μm. **d** A microscopic image of R/G double QD patterns under UV light. **e** A microscopic image of RGB pixels for the QD-integrated μ-LED arrays. **f** Schematic of the QD color conversion process. **g** PL spectra of the green/red QD layers. **h** Absorbance of the blue light from the LED as a function of QD film thickness. The absorbance is measured by subtracting the transmitted blue light power from the incident blue light power. **i** Normalized light power intensities of the absorbed blue light, emitted green light, and transmitted blue light as a function of the green QD film thickness. The EQE saturates as the QD thickness increases. **j** EQE and IQE as a function of the QD film thickness for both green and red QDs.

As presented in Fig. 4a, a wafer-scale full-color micro-LED display with 5 μm pixels was successfully fabricated on a new silicon substrate. The integrities of the polychromatic QD arrays (without LEDs) with 10 and 5 μm-pixel sizes are presented in Fig. 4b, c, respectively. Figure 4d presents images of both green and red QDs that were consecutively patterned in a two-step process. A full RGB profile was achieved by using the red/green QDs as color-converting layers that down-converts the blue emission from the GaN micro-LEDs to the corresponding red/green emissions (Fig. 4e). The final LED array had a 20 μm RGB pitch, and the red/green QD color conversion layer was bonded on top of the blue LEDs. First, the QDs were coated and patterned on a thin glass layer (500 µm thick) with lithography-level precision using the elastomer-assisted dry-patterning process. Then the glass plate was positioned with a micro-positioner using a microscope and bonded with an epoxy layer. The size difference of the red/green vs. blue pixel was attributed to the distance gap between the GaN LED and the color conversion layer.

There would be three light components one needs to consider to understand the optical properties of the QD layer: transmitted (leakage), emitted, and absorbed light. Figure 4f shows the schematic of such a QD color conversion process. The transmitted and the scattered blue light would constitute the blue light leakage and the down-converted light would constitute the emitted green light. Also, some of the blue light would be absorbed (Fig. 4f).

To understand the relationship between the QD layer and light conversion efficiency in detail, several experiments were carefully conducted (Fig. 4g–j). Some of the terms (e.g., quantum efficiencies, absorbed light, and light power) are defined in the Method section. Figure 4g presents the corresponding PL spectra and luminance values as a function of the QD layer thickness. From the initial inspection, although the thicker QD layer was correlated to increased light conversion, the conversion efficiency seems to saturate as the layer thickens. As presented in Fig. 4i, light absorbance increases (and light leakage decreases) with film thickness. Importantly, this trend is not linear and quickly saturates. The combined response of the absorbance, light penetration, and various secondary effects is shown as external quantum efficiency (EQE) (Fig. 4I, j). As presented in Fig. 4i, the EQE values increased up to a certain QD thickness and saturated. This result suggests that the blue light that is not converted is decayed to other forms, such as scattering, reflection, and absorption. Due to Stoke’s shift, the phenomenon of self-absorption and reabsorption among QDs is more pronounced as the film thickness increases^76^. A thicker film would also lead to a decrease in transmittance^77^ and the fluorescence intensity of the emitted green/red light. Moreover, even if the QD layer thickens and blue light leakage decreases, some of the blue light will also not reach the top QD layer, thus demonstrating a saturation of the light-emitting efficiency. As shown in Fig. 4j, the EQE would quickly saturate below the internal quantum efficiency (IQE) level as the QD layer thickness increased. Regarding the EQE of previous reports, Chen et. al. report EQE values of 51% and 38%, for their 6 µm-thick red and green QD layers, respectively^78^. Lee et al. also report an EQE of 25–30% for green QD between 4 and 6 µm thick, and an EQE of 34–38% for red QD between 4 and 6 µm thick^79^. These values are very similar to EQE of 32.5–37% for green QD between 3 and 5.5 µm thick, and an EQE of 37–38% for green QD between 4.5 and 5.5 µm thick shown in our work (Fig. 4j). Owing to the limited spot sizes of the equipment, the quantum efficiencies of the QD layers were measured with a non-pattern QD film on top of the GaN LED array. More details of the experimental process involved in Fig. 4 are included in the Method section.

At the mass-production stage, the QDs would be integrated on a planarized surface of GaN LEDs coated with peripheral layers (e.g., distributed Bragg reflector (DBR) layer or a black matrix layer in between mesas) to reduce optical crosstalk and improve the color purity. While the main focus of this work is the experimental demonstration of color conversion and high-resolution patterning of QDs, the effect of a DBR layer on color purity is quite important^80,81^. Hence the effect of using a DBR on the QDs and LEDs was studied and shown in Supplementary Fig. S8 and S9. First, an optimal combination of alternating thin films was found with an optical simulation program (Essential Macleod, Methods). Then DBR layers were fabricated with alternating layers of SiO_2_ and TiO_2_ on a separate glass substrate. The DBR was highly reflective in the blue spectral region, and the chromaticity chart in Fig. S8c shows high spectral purity of the R, G, B colors with the usage of a DBR structure. Notably, the fwhm values of the R,G,B spectrum are similar to those of a comparable work that uses black and grey PR matrices, which affirms the importance of peripheral technologies^78^. In Supplementary Fig. S9, the chromaticity coordinates with and without the DBR are compared to demonstrate the effectiveness of DBR in reducing the optical crosstalk and improving color purity.

## Discussion

This work is a culmination of several techniques for achieving high-resolution GaN LED arrays with QD-based color conversion. The main achievement of this study is twofold. First, a wafer-scale, self-emissive, 5 μm-sized GaN micro-LED arrays were integrated on a foreign silicon substrate. The bond-first, pattern-later technology and the thin eutectic bonding layer used in this work enabled lithography-level alignment with higher accuracy compared to the flip-chip bonding technique. The second achievement is the integration of a QD-based color conversion layer with lithography-level resolution using solvent-free processes. This was accomplished by carefully controlling the surface energy and utilizing elastomeric topographical masks. Although this is significant progress for future displays required in HMDs, there is sufficient room for improvement in terms of fill factor and light leakage. The application of peripheral technologies such as a DBR that improve these factors will further enhance the performance in the future. With such advancements, this technology, which adopts QDs, GaN, and silicon electronics in a single platform, will pave the way for near-eye displays suitable for forthcoming novel applications.

## Methods

### Elastomeric mask preparation and patterning of QDs

A 996 kg/mol PMMA mixed with toluene of 3-wt/vol% concentration, resulted in a 560 nm-thick film that was subsequently baked on a hotplate at 120 °C for 10 min. PMMA is coated first because the polarity of the photoresist solvent is not suitable for coating the FOTS surface-treated glass substrate. Subsequent photolithography was conducted with a contact aligner. The PMMA exposed by patterned PR was etched with O_2_ plasma. The FOTS is removed simultaneously by the O2 plasma (Supplementary Fig. S4a). Then the remaining PR and the glass surface were treated with CF4^82^-based RIE. Spin coat QD (Chem-E Co.) on a glass substrate with PMMA/PR patterns and cure for 2 min at 100 °C and 10 min at 180 °C on a hotplate. Separate the PMMA/PR and the glass substrate with cured QDs using adhesive polymer (Fig. 3b, c). This leaves only the QD pattern on the glass substrate (Fig. 3d–g) because the weakly adhesive PMMA/PR is separated and the adhesive QD remains. During the whole process, the QD does not come into contact with any liquid.

### Measurement of quantum efficiencies

The measurement of quantum efficiencies was conducted using a quantum efficiency measurement system (QE-1100, Otsuka Electronics). We have clarified the terms we used as shown below.

1. EQE(%) = blue absorption(%) × IQE
2. Blue absorption = (Total blue light power − blue light power leakage) / Total blue light power
3. IQE (i.e., conversion efficiency) = Converted red or green light power/(total blue light power − blue light power leakage)

Power values are extracted by summation of the relevant spectrum (e.g., blue = 430–490 nm)

### Distributed Bragg Reflector (DBR) implementation

The target total thickness of the DBR structure was 605 nm and alternating films of SiO_2_ and TiO_2_ were used. The thin-film stack simulation for the DBR was conducted with the Essential Macleod tool. The QD measurement was conducted with a spectrometer (AvaSpec-ULS3648, Avantes), a probe station, and a source meter (Keithley 2602B). The measurement was set to a wavelength of 300–800 nm and the current values ranged from 0.1 μA to 10 mA. From the spectral data, the color coordination for the CIE chromaticity chart was deduced. First, the tristimulus values X,Y,Z were determined by the integral of the product of spectrum power and the color matching function for the corresponding red, green, blue spectrum. Then the CIE chromaticity coordinates were derived from the relationship Cx = X/(X + Y + Z), Cy = Y/(X + Y + Z), and Cz = Z/(X + Y + Z). To prevent manual error, we used the Chromaticity diagram program from Origin software to derive the chromaticity coordinates.

## Supplementary information

Supplementary Information

## Data Availability

The data that support the findings of this study are available from the corresponding author upon reasonable request.
