# Supplementary material for: Quantum dot-integrated GaN light-emitting diodes with resolution beyond the retinal limit
Source: Nat Commun. 2022 Apr 6;13:1862. doi: 10.1038/s41467-022-29538-4 (PMC8986835; doi:10.1038/s41467-022-29538-4)
Supplement: Supplementary file 1 — Supplementary Information [file 41467_2022_29538_MOESM1_ESM.pdf]

Supplementary Information

Quantum dot-integrated GaN light-emitting diodes with resolution beyond the retinal limit

Junho Bae[1*], YuSeop Shin[1*], Hyungyu Yoo[1,3], Yongsu Choi[1,3], Jinho Lim[1,3], Dasom Jeon[1,3], Ilsoo Kim[2], Myungsoo Han[2], and Seunghyun Lee[1,3§].

[1]*Department of Electrical Engineering, Kyunghee University, 17104, Rep. of Korea*
[2]*LG Display Research and Development Center, Seoul 07796, Rep. of Korea*
[3]*Department of Electronics and Information Convergence Engineering, Kyunghee University, 17104, Rep. of Korea*

Supplementary Discussion 1 | Perceived speed of a display technology

It is generally known that inorganic semiconductors such as GaN have faster electrical response due to its higher carrier mobility compared to that of an organic materials. However, there are other overlooked components in the display system that may be important in determining the perceived total response speed [18].

The response time of mLED/µLED/OLED chips is generally several orders faster than that of liquid crystals (LCs). However, we cannot conclude that mLED/µLED/OLED emissive displays always provide a much smoother visual experience than LCDs. MPRT (motion picture response time) is often used to quantify the perceived visual response.

MPRT is related to the pixel response time (τ) and the frame rate (T_f=1/f).

When $\tau \ll T_f$, MPRT is mainly determined by T_f, so a high frame rate helps to reduce the MPRT.

$$\text{MPRT} = \sqrt{\tau^2 + (0.8T_f)^2}$$

For example)

f=60fps, LCD(Response Time : 2 ms) → MPRT (13.5 ms) Liquid crystal change time + phosphor reaction time.

f=60fps, µLED/OLED (Response Time : order of µs/ns) → MPRT (13.3 ms)

Currently, sub-millisecond MPRT is possible with OLED/micro LED.

It is also possible for LCD to reach such MPRT with the reduction of DR value. DR (Duty ratio) is the ratio between the light emission time and the frame time. However, the decreased DR is a trade-off since it also decreases the LCD luminance.

As a conclusion, there has been many reports on how µLED have superior electrical response speed. However, the perceived response speed is a combinational effect of several factors in a display system. Hence, it may be ill-advised to conclude that µLEDs always result in faster response speed compared to other technologies.

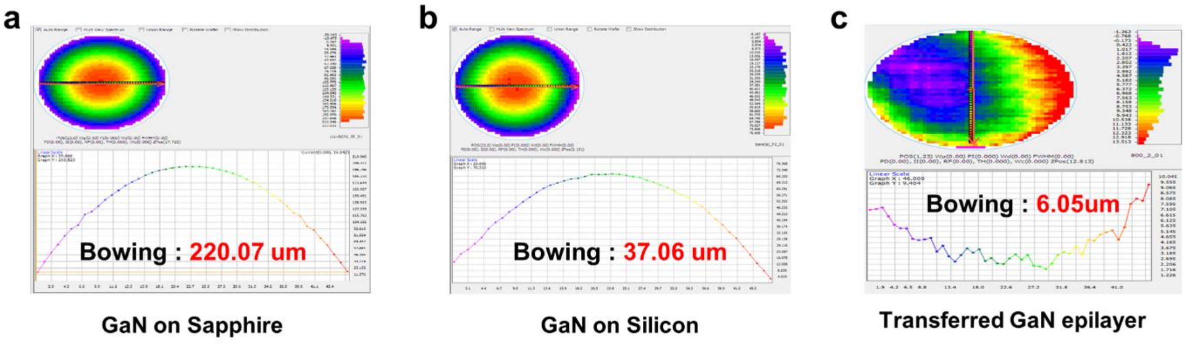

Supplementary Figure S1 | Topographical mapping result of GaN on sapphire and GaN on silicon wafers All wafers are 4inch in diameter a GaN on sapphire wafer mapping result. **b** GaN on silicon wafer mapping result **c** GaN epitaxial layer transferred to a second substrate.

Supplementary Note 1 | Wafer bowing of 4inch Gallium nitride wafer

Supplementary Figure 1a shows the wafer bowing (220.7 μm) from the center to the edge of the GaN on the sapphire wafer. Such a level of bowing makes lithography extremely difficult. This is more pronounced in this work since the individual LED size is only 5 ×5 μm^2 and alignment is crucial. To reduce the wafer bowing, an epitaxial layer transfer method was devised. Figure 1c shows significantly reduced bowing level (~6 μm) after the GaN epitaxial layer was transferred to a foreign silicon wafer.

Supplementary Note 2 | Epitaxial layer transfer method

a

Wafer	Metal layer	1st Thickness(nm)	2nd Thickness(nm)
GaN	Ti	20	20
	Ni	100	100
	Sn	300	700
	Ni	3	3
Si IC	Ni	3	3
	Sn	300	700
	Ni	100	100
	Ti	20	20

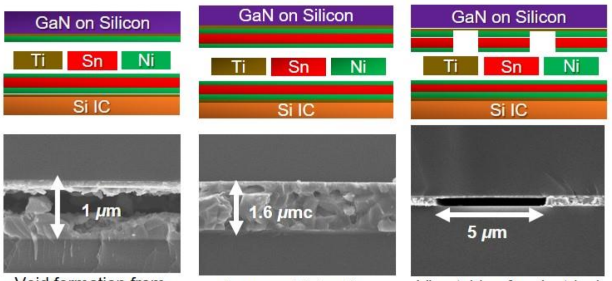

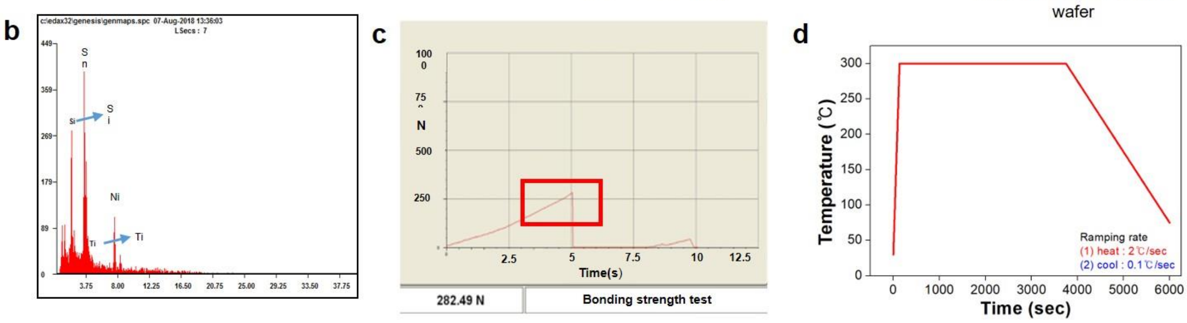

Supplementary Figure S2 | Low temperature, thin layer bonding process. a Bonding recipe and FE-SEM images. Via etching to that is aligned to the bottom wafer for electrical connection is clearly shown **b** EDX measurement of Sn-Ni alloys. **c** Shear stress test **d** Temperature profile of Ni-Sn TLP bonding process.

The epitaxial transfer method requires optimization of wafer bonding and substrate removal process. Considering a GaN epitaxial layer thickness of 3 μm and a pixel size of 5 μm, the bonding layer thickness should be quite thin. Therefore, a bonding recipe with a low thickness requirement and sufficient bonding strength is required. In addition, care must be taken to set the bonding temperature below 400 °C for CMOS compatibility. Therefore, a transient liquid bonding (TLP) bonding process using Ni/Au is a possible method. However, owing to the high cost of Au, Au was replaced with Sn and similar performance was confirmed. TLP bonding is a bonding process that enables alloy formation between two metals and the alloy forming temperature (300°C) is typically lower than the melting temperatures of individual metals. Figure 2a shows several bonding recipes attempted in this study, their schematic, and the relevant FE-SEM image. 1st bonding recipe is partially incomplete and the 2nd bonding recipe shows perfect bonding. It also shows that bonding is possible even with patterned metal. Figure 2b is an analysis of Ni/Sn TLP bonding through EDX profile. The profile shows that Ni/Sn alloy has been successfully formed. Figure 2c is the shear stress test result, and Figure 2d shows the temperature profile during the bonding process.

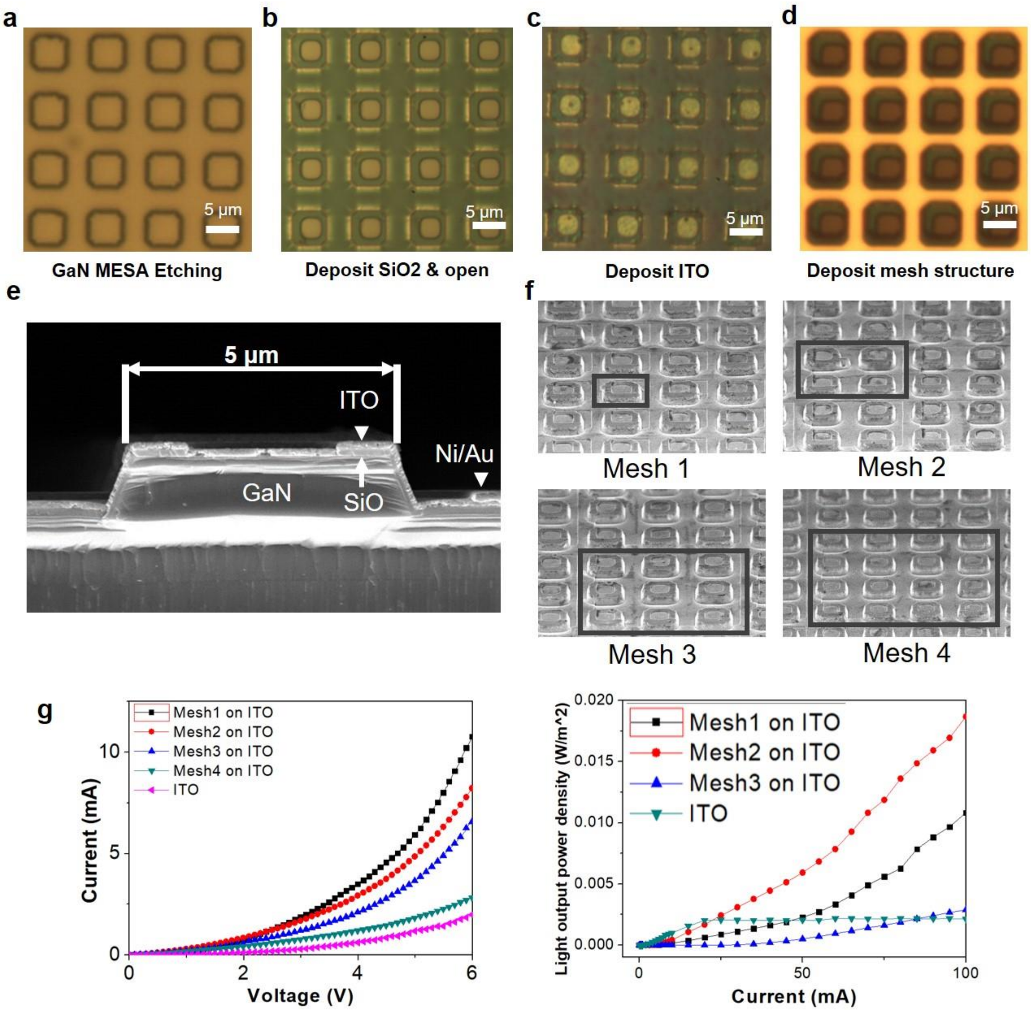

Supplementary Figure S3 | microscope image of fabrication process and FE-SEM image of micro-LEDs. a image of GaN mesa structures. **b** image of SiO_2 opening. **c** image of ITO deposited mesas. **d** image of hybrid mesh structure. **e** FE-SEM image of epitaxtial transferred micro-LED. **f** FE-SEM image of hybrid mesh structures,with grid density of 1x1, 2x2, 3x3, and 4x4, respectively. **g** Collective current as a function of voltage and light output power density as a function of collective current. Both plots are from 10000 pixel panel.

Supplementary Note 3 | Fabrication procedure of epitaxial layer transferred μ-LED

The process is as follows. The wafer bonding and substrate removal described above are first performed. In this process, the GaN epitaxial layer consisting of p-GaN, MQWs, n-GaN, undoped GaN, and the buffer is attached to the second silicon wafer using the bonding process. Then, inductively coupled plasma (ICP) etching is performed to etch 1.5 μm of unnecessary buffers, and undoped GaN. 5 μm -sized pixels are formed through photolithography and BCl_3/Cl_2 based ICP etching. For electrical isolation, 100nm SiO_2 is deposited on the mesa structure using PECVD. Next, SiO_2 is opened by using photolithography and reactive ion etching. For electrical connection, ITO and hybrid mesh are deposited through the e-beam evaporation and lift-off process. The side profile of an individual micro-LED is shown in Figure 3e. Figure 3f shows the FE-SEM tilted images of the hybrid mesh structures.

a

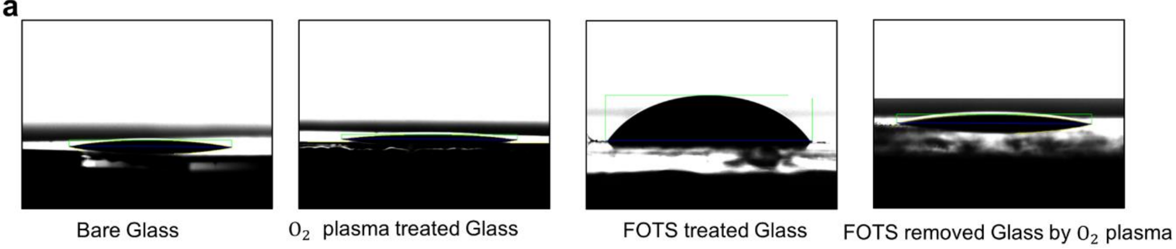

Measurement result				Unit : deg.
Sample	1st	2nd	3rd	Average
Bare Glass	7.60	7.40	7.20	7.40
O2 plasma Glass	4.07	4.13	4.69	4.30
FOTS Glass	37.89	36.70	41.50	38.70
remove FOTS Glass	8.89	8.85	7.94	8.56

b

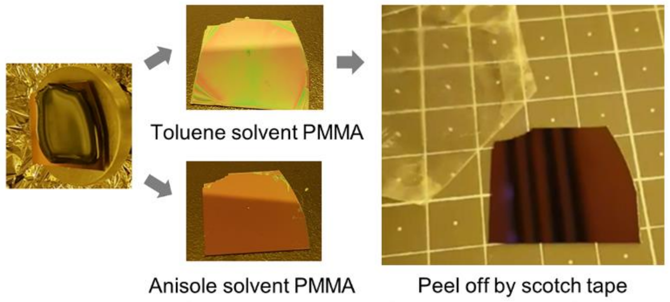

Solvent	Formula	Density (g/mL)	Relative polarity[2]
Toluene	C_7H_8	0.867	0.099
Chlorobenzene	C_6H_5Cl	1.106	0.188
Anisole	C_7H_8O	0.996	0.198

Supplementary Figure S5 | contact angle test and PMMA adhesion test comparing different solvents. a The test showing different contact angle of each state of the glass substrate. FOTS treated surface exhibit hydrophobic property. **b** Due to the polarity of the pmma solvent, toluene solvent is suitable for PMMA spin coating. PMMA layer can be physically detached with a small force.

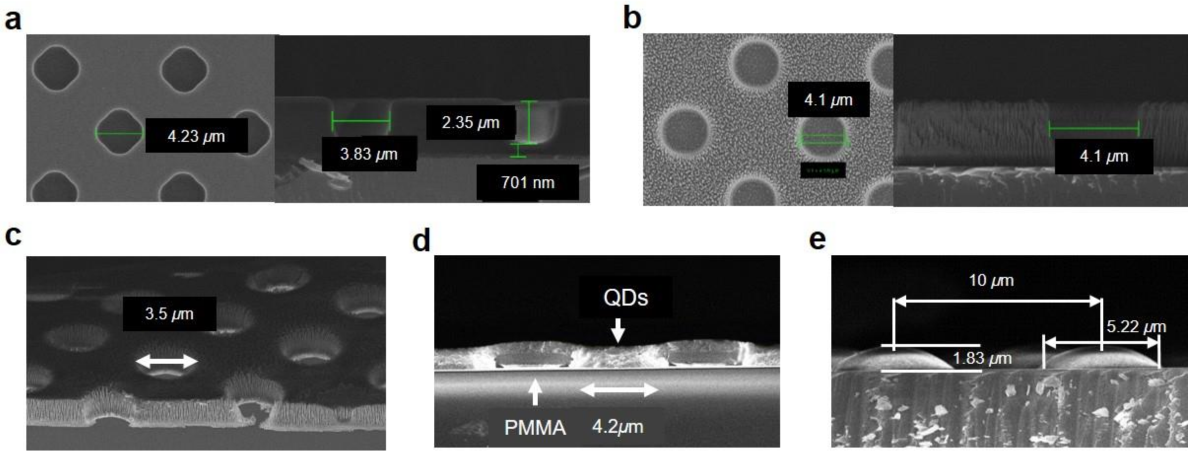

Supplementary Figure S4 PMMA and quantum dots patterning process. a FE-SEM image of PR pattern on PMMA by photolithography process. **b** FE-SEM image of patterned PMMA using O_2 plasma etching. **c** FE-SEM image of patterned PMMA layer separated from the substrate. **d** FE-SEM image of quantum dot coating on the patterned PMMA. **e** FE-SEM image of remaining quantum dot patterns after the peel-off process.

a

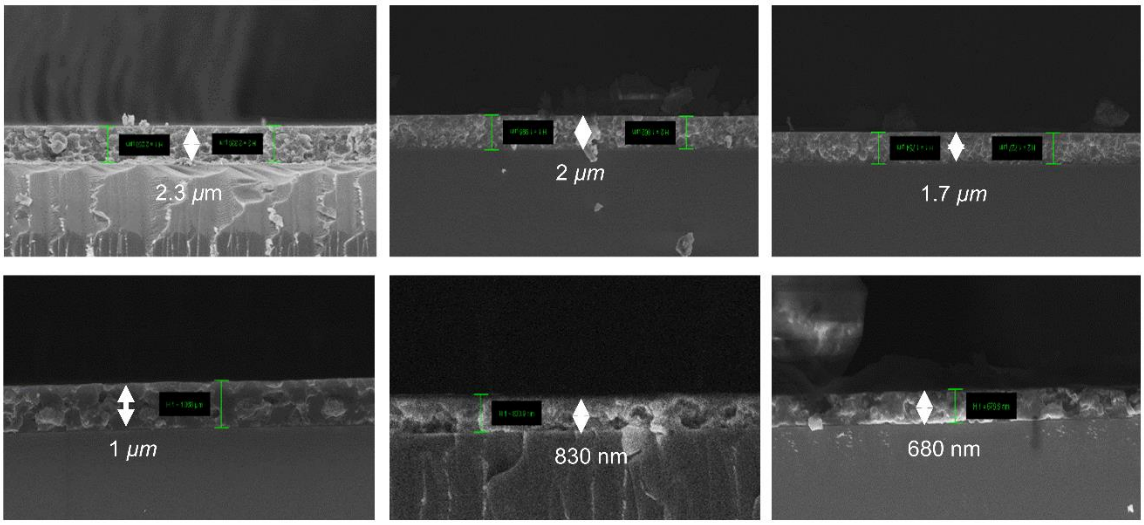

b

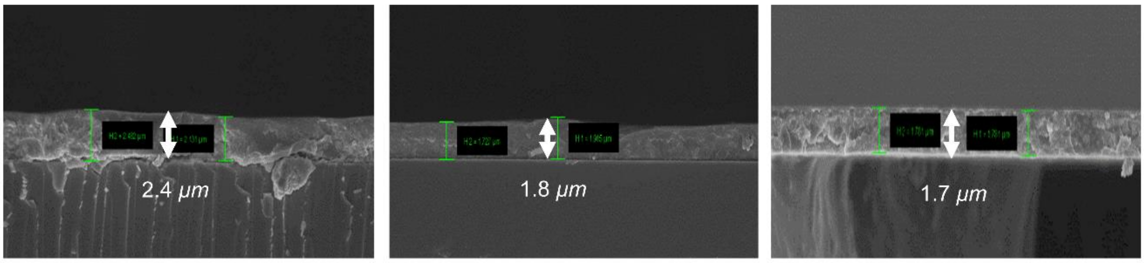

c

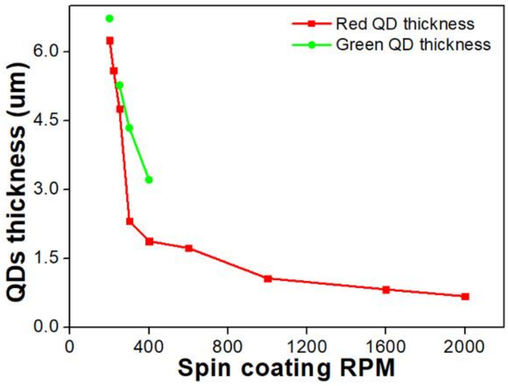

Supplementary Figure S6 Control of quantum dot films thickness. a Red QD films thickness with varying spin coating speed **b** Geen QD films thickness with varying spin coating speed **c** QD films thickness as a function of spin speed.

The effect of baking time and temperature on the QD quality

PR RPM	1500rpm	->	->	->	->	->
RIE time (O2)	150w, 90s	->	->	->	->	->
RIE time (CF4)	150w, 90s	->	->	->	->	->
QD rpm	1000rpm	->	->	->	->	->
**QD baking**	**90℃ 2min 180℃ 0min**	**90℃ 2min 180℃ 1min**	**90℃ 2min 180℃ 2min**	**90℃ 2min 180℃2.5m**	**90℃ 2min 180℃ 3min**	**90℃ 2min 180℃ 4min**
QD 현미경	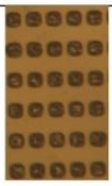	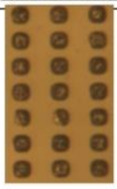	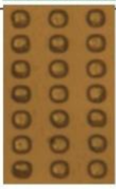	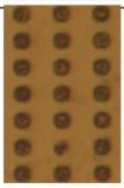	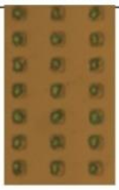	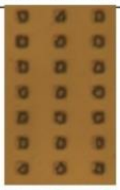
QD 현미경 UV	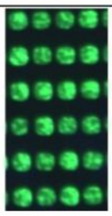	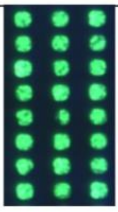	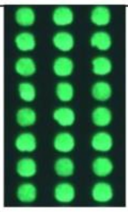	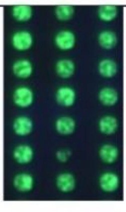	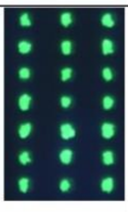	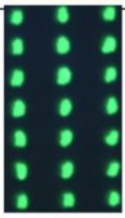

Correlation between QD thickness and QD shape, yield.

	QD Soft bake	QD Soft bake	QD Soft bake	QD Soft bake	QD Soft bake
PR RPM	1500rpm	->	->	->	->
RIE time (O2)	150w, 90s	->	->	->	->
RIE time (CF4)	150w, 90s	->	->	->	->
**QD rpm**	**400rpm**	**600rpm**	**800rpm**	**1000rpm**	**1200rpm**
Microscopic image of patterned QDs	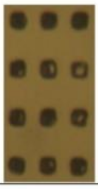	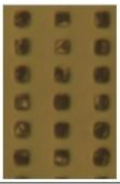	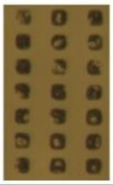	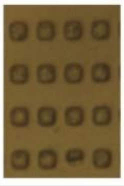	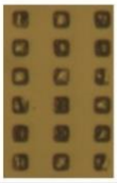
Microscopic image of patterned QDs with UV light	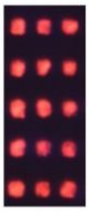	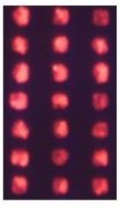	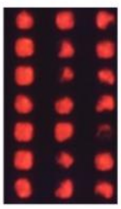	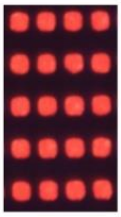	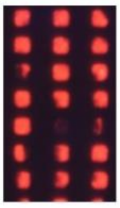

Supplementary Figure S7 The effect of different recipes on the QD shape and yield. a. The effect of baking time and temperature on the QD quality **b.** Correlation between QD thickness and QD shape,

The QD baking temperature and time was found to be crucial in forming a fully shaped QD patterns. The top figures show how green QDs exhibit weak adhesion behavior when it is not baked fully. Interestingly, if the baking time exceeded an optimal time, the edge of the QD patterns were eroded after the elastic mask removal. We believe this is from side edge of the QD layer being attached to the elastic double layer mask. As written the manuscript, the adhesive force of the PMMA sidewall was also quite high for the QDs to leave a dependable layer attached to

the bottom substrate after the peel-off process. As fluoropolymers have weaker adhesive properties, CF4 treatment was found to be highly effective in increasing the yield and thickness of the QD patterns by lowering the side adhesiveness of the elastomeric mask. The balance between the adhesive force of QD to the bottom substrate and the side mask was found to be critical.

Such an effect was also found with spin rpm of the QD. We found out that the spin speed is not the sole parameter that determines the final QD pixel thickness. It was the combination of spin RPM, the elastomeric mask thickness, and the size of the QD patterns that determined the final thickness. As shown in the bottom figure above, if the spin speed was too slow, the QD would be too thick and during the baking process, the QD layer would not separate and yield will be low. If the spin speed was too high, the QD would not fill up the trench properly and there would be missing pixels. Hence, it is not just the spin speed that determines the final QD pixel thickness, but also the ratio between the QD layer thickness and the double layer elastomeric mask height. The adhesiveness of the bottom substrate and the side of the elastomeric mask also plays a part.

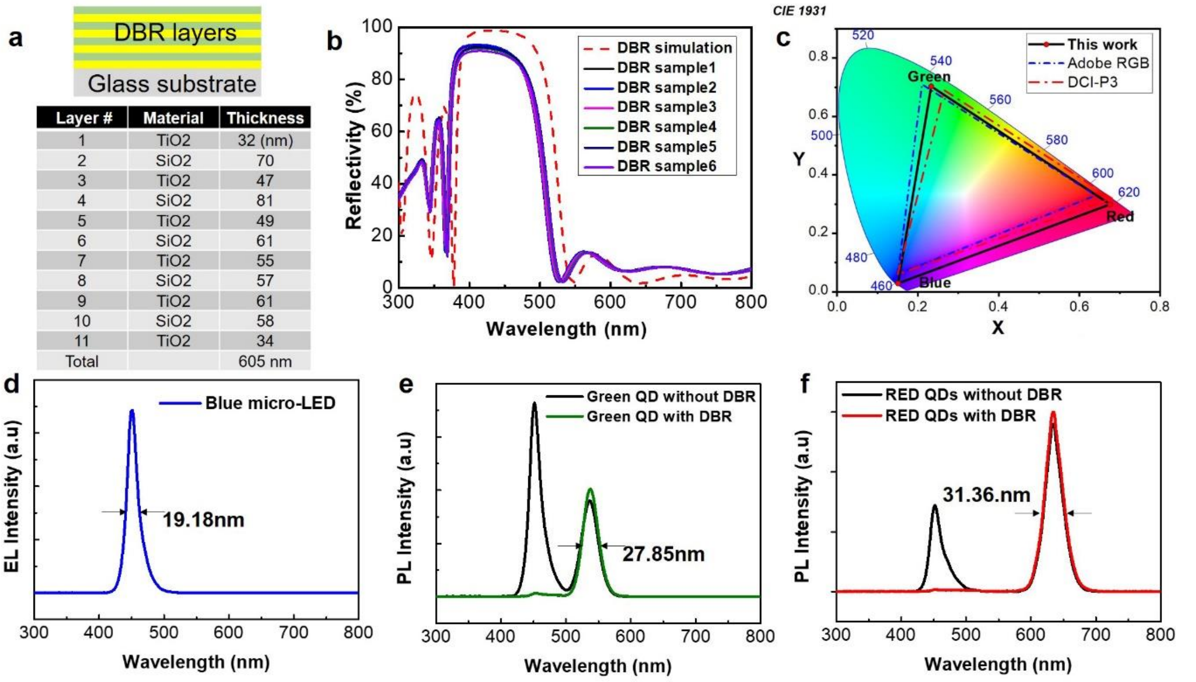

Layer #	Material	Thickness
1	TiO2	32 (nm)
2	SiO2	70
3	TiO2	47
4	SiO2	81
5	TiO2	49
6	SiO2	61
7	TiO2	55
8	SiO2	57
9	TiO2	61
10	SiO2	58
11	TiO2	34
Total		605 nm

Supplementary Figure S8 The effect of DBR on QD color spectral purity. a. The structure of the multi-layered DBR fabricated on a glass substrate. The target thickness of the DBR was 605nm to match LED size **b.** A comparison between simulated DBR results and the fabricated samples. The DBR is highly reflective in the blue spectrum. **c.** CIE chromaticity diagram of the red, green, blue spectrum with experimental results using DBR substrate on top of the fabricated blue LED, red QD on LED, and green QD on LED, respectively. The final R,G,B coordinates are R : 0.67554, 0.2992 ; G : 0.23287, 0.70322; B : 0.1507, 0.02907, respectively. A comparison with Adobe RGB and DCI-P3 is also shown. DCI/P3 is a common RGB color space for digital movie projection from the American film industry. This corresponds to DCI-P3 color gamut ratio of 109.09% and adobe RGB color gamut ratio of 109.7% in the CIE 1931. **d,e,f** Spectral intensities of blue (LED only), green (QD with LED), red colors (QD with LED) with and without DBR, respectively. The full-width-at-half maximum values are also shown.

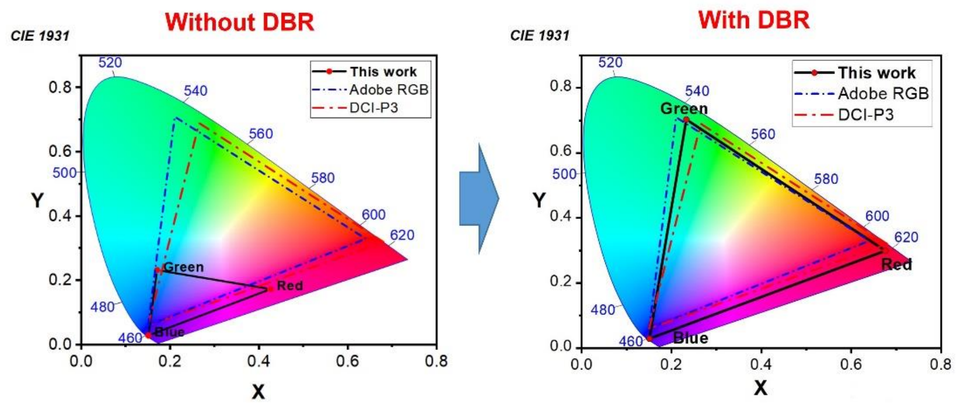

Supplementary Figure S9 The chromaticity coordinates with and without DBR on a QD+LED structure. The enlarged triangle (solid line) in the right figure exhibits a dramatic reduction on blue light induced optical cross talk and show improved color purity.
